# Towards a better understanding between non-Muslim primary care clinicians and Muslim patients: A literature review intended to reduce health care inequities in Muslim patients

**DOI:** 10.1016/j.hpopen.2023.100092

**Published:** 2023-03-24

**Authors:** Jeffrey K King, Alexander Kieu, Marwan El-Deyarbi, Noof Aljneibi, Saif Al-Shamsi, Muhammad Jawad Hashim, Linda Östlundh, Kate Ellen King, Renee Houjintang King, Moien AB Khan, Romona Devi Govender

**Affiliations:** a Department of Family Medicine, College of Medicine and Health Sciences, United Arab Emirates University, Al Ain, Abu Dhabi, United Arab Emirates; b Home Based Primary Care, Division of Extended Care and Geriatrics, Department of Veterans Affairs, Greater Los Angeles area, CA, USA; c Kanad Hospital, Al Ain, Abu Dhabi, United Arab Emirates; d Ambulatory Health Services, Abu Dhabi, United Arab Emirates; e Department of Pharmacology, College of Medicine and Health Sciences, United Arab Emirates University, Al Ain, Abu Dhabi, United Arab Emirates; f Emirates Center for Happiness Research, United Arab Emirates University, Al Ain, Abu Dhabi, United Arab Emirates; g Department of Internal Medicine, College of Medicine and Health Sciences, United Arab Emirates University, Al Ain, Abu Dhabi, United Arab Emirates; h Ӧrebro University, Ӧrebro, Sweden; i Scripps College, Claremont, CA, USA; j Academic Family Medical Center, Ventura County Family Medicine Residency Program, 300 Hillmont Ave, Building 340, Suite 201, Ventura, CA, USA; k Health and Wellness Research Group, Department of Family Medicine, College of Medicine and Health Sciences, United Arab Emirates University, Al Ain, Abu Dhabi, United Arab Emirates; l Primary Care, NHS North West London, London TW3 3EB, United Kingdom

**Keywords:** Islam, Muslim, Religion, Discrimination, Outcomes, Primary care

## Abstract

Although Muslims are a growing population within many non-Muslim countries, there are insufficient Muslim clinicians to care for them. Studies have shown that non-Muslim clinicians have limited knowledge and understanding of Islamic practices affecting health, which may lead to disparities in the quality of healthcare delivery and outcomes when caring for Muslim patients. Muslims come from many different cultures and ethnicities and have variations in their beliefs and practices. This literature review provides some insights which may strengthen therapeutic bonds between non-Muslim clinicians and their Muslim patients resulting in improved holistic, patient-centered care in the areas of cancer screening, mental health, nutrition, and pharmacotherapy. Additionally, this review informs clinicians about the Islamic perspective on childbirth, end of life issues, travel for Islamic pilgrimage, and fasting during the month of Ramadan. Literature was sourced by a comprehensive search in PubMed, Scopus, and CINAHL along with hand screening of citations. Title and abstract screening followed by full-text screening excluded studies including less than 30% Muslim participants, protocols, or reporting results deemed irrelevant to primary care. 115 papers were selected for inclusion in the literature review. These were grouped into the themes of general spirituality, which were discussed in the Introduction, and Islam and health, Social etiquette, Cancer screening, Diet, Medications and their alternatives, Ramadan, Hajj, Mental health, Organ donation and transplants, and End of life. Summarizing the findings of the review, we conclude that health inequities affecting Muslim patients can be addressed at least in part by improved cultural competency in non-Muslim clinicians, as well as further research into this area.

## Highlights

• This paper updates a review of the literature on this topic, including papers published as recently as 2021.
• The writing team includes a balanced mixture of Muslim and non-Muslim clinicians with experience in caring for Muslim patients from a variety of disciplines: primary care, hospital care, pharmacy, and mental health.
The majority of the team has practiced in both Muslim and non-Muslim majority countries.
• Due to the relatively low amount of research in investigating the inequities experienced by Muslim patients in non-Muslim majority settings, some of the studies are in subsets of Muslim patients, particularly refugee and immigrant populations. Caution is needed in the interpretation and application of the results of these studies, which is pointed out in the text.

## 1 Introduction

Disparities in health care adversely impact patient-centered care and threaten the selfless nature of the practice of medicine. Healthcare inequities have attracted growing attention in the last decade, as it has become recognized that certain populations have poorer health outcomes than others. These disparities are multifactorial, occurring at the junction of economic, legal, public health and other social determinants.[1].

Clinicians, particularly those in primary care, seek to promote the delivery of holistic, patient-centered continuity care[2], and thus are uniquely positioned to address healthcare disparities, both while caring for individual patients as well as at the level of healthcare systems[3], [4], [5]. The biopsychosocial model conceptualizes the care of the whole person using a framework that acknowledges and addresses each individual’s biological, social, psychological, and spiritual strengths and needs.[6], [7] When a clinician and patient are from discordant faith backgrounds, a clinician’s lack of familiarity with a patient’s worldview can adversely impact rapport and communication. This can negatively affect patient adherence to a care plan.[8] Conversely, familiarity and awareness with each aspect of a patient’s life can have powerful therapeutic effects.[9], [10], [11] Although certain ethical and professional boundaries must be respected[12], significant proportions of patients in multiple studies have agreed that they prefer clinicians inquire about their spirituality in order to understand them both as a person and as a medical decision-maker;[13], [14] however, few report that their clinicians had done so.[15] Spiritual assessment can incorporate formal, validated screening tools such as the FICA (Faith, Importance of spirituality, Community, and Action)[16], [17] or can be respectful, patient-centered informal inquiries or noticing a patient’s cues or what they are wearing[18].

The professional practice of medicine requires the recognition of the primacy of the patient, which marks medicine as a caring and selfless endeavor. This stretches all the way back to the original Hippocratic Oath, which states “Whatever houses I may visit, I will come for the benefit of the sick, remaining free of all intentional injustice.”[19] In seeking this “benefit”, clinicians should educate themselves about the cultural practices of their patients with the goal of reducing implicit bias and providing high quality, culturally competent individual care[20], [21], [22], as well as to advocate for their patients at the systemic level.[23].

There are over one billion Muslims worldwide, and Islam is the third most common religion in the United States. Certain areas of the country are home to large Muslim communities. By 2050, the number of Muslims in the United States is projected to double, making Islam the second-largest religion in the country, if current trends continue.[24] However, this rapid increase is not being matched with a corresponding increase in either the number of US-trained Muslim physicians or International Medical Graduates from Muslim-majority nations[25], which makes the probability of a non-Muslim clinician treating a Muslim patient increasingly likely. Providing cross-cultural medical care presents certain potential challenges and rewards[26], [27], [28], and a non-Muslim clinician’s level of awareness of Muslim practices will directly impact the delivery of high-quality patient care to this population of patients.[29], [30] In particular, the literature regarding the relationship between Muslim patients and health care inequities is limited, but surveys and qualitative interviews have identified several mechanisms by which identified inequities are mediated, which include awareness and access to care, differing interpretations of health, conflict between Islamic values and standard treatments, and perceived discrimination due to a lack of cultural understanding and accommodation.[31], [32], [33], [34], [35], [36] One study found that health care utilization fell after the 2016 presidential campaign and subsequent implementation of restriction of immigration from Muslim-majority countries, even among people from countries not targeted by that ban.[37].

Islam calls for religious practices that affect multiple areas of life, many of which have potential clinical significance. Although Islam is thought by many unfamiliar with the religion to be a homogeneous bloc, significant variations in belief and practice exist which may be additionally be influenced by personal, familial, ethnic and other factors.[38], [39], [40] This article, therefore, is not intended to be an exhaustive or definitive authority on the beliefs and practices of every Muslim but rather an introduction to commonly encountered situations that healthcare providers should be aware of in treating Muslim patients in a culturally sensitive way. For those with a lack of familiarity with a patient’s personal practices and beliefs, a learning attitude rather than an assuming attitude should be adopted. Open-ended questions such as, “Can you tell me more about how…?” or “I’ve heard that Muslims […]. How does that affect how I care for you as a patient?” can lead to enriching and rewarding conversations.[41] This approach is consistent with the concept of ‘cultural humility’ which over the past two decades has added further nuance to the accepted goal of ‘cultural competence’ by adding the recognition that each of us has a limited but modifiable capacity for cultural understanding that can be expanded through a life-long learning process (there are varying degrees of cultural competence as well as diversity among individuals within cultural groups).”[42].

## 2 Methods

A comprehensive search in PubMed, Scopus and CINAHL was performed in January 2022 to support the inclusion of studies in the literature review. The search terms for “primary care” with synonyms were combined with search terms for “Islam”, “Muslim” etc. The search was conducted with a combination of the search fields “title”, “abstract” and MeSH/Subject Headings for the best possible results. A filter for English language and publication year 1995 to the search date was applied. Reproducible search strings for all three databases are appended (see appendix 1).

**Fig. 1:**
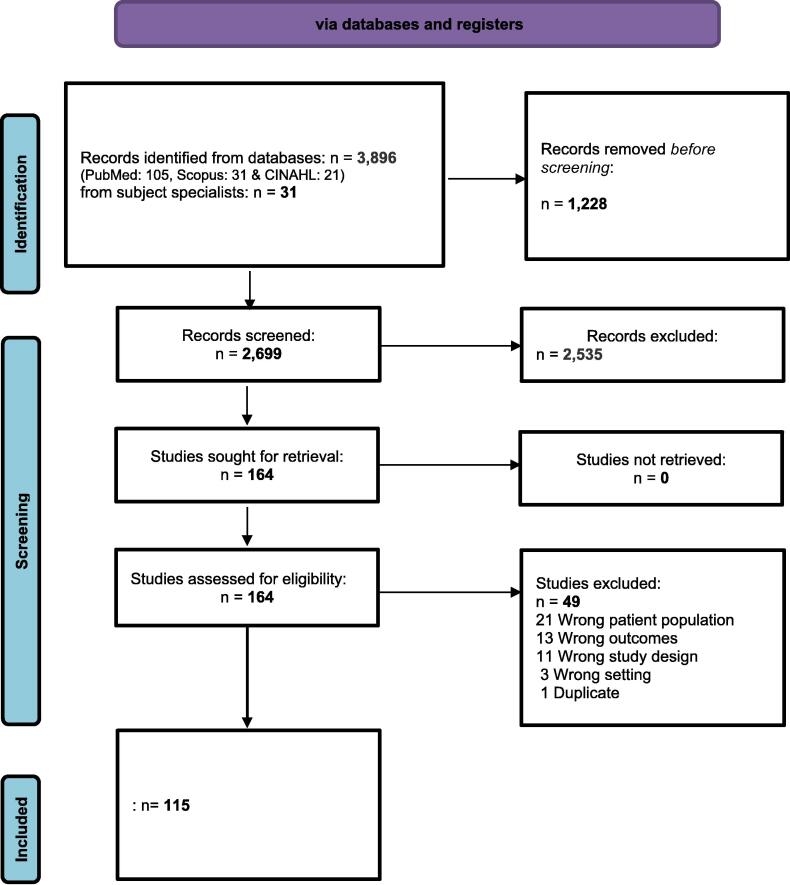
PRISMA 2020 flow diagram for literature review. *From:* Page MJ, McKenzie JE, Bossuyt PM, Boutron I, Hoffmann TC, Mulrow CD, et al. The PRISMA 2020 statement: an updated guideline for reporting systematic reviews. BMJ 2021;372:n71. https://doi.org/10.1136/bmj.n71.

To support a systematic and transparent selection of evidence for this literature review, the study screening and selection follows the PRISMA guidelines[43] and was carried out using the review software Covidence[44]. All records identified in the database search were screened for eligibility in two steps: title/abstract and full text. In addition, hand screening of the reference lists in the included studies were conducted together with citation snowballing in the databases to identify relevant websites, book chapters and other gray materials. Included studies addressed issues relevant to the primary care of Muslims as adjudged by the writing team. Studies were excluded if they did not include at least 30 % Muslim participants; were protocols, opinions, or letters to the editor; or reported results deemed irrelevant to primary care. Finally, subject specialists with experience in caring for Muslim patients in primary care were consulted for additional literature suggestions. A PRISMA flowchart was used to document both the automatic and the manual collection, screening, and selection of literature (see Fig. 1). The academic status of the final selected papers published in open access journals was checked with the help of Cabell’s Predatory Reports.[45].

## 3 Results

In total, 2,699 unique studies were identified in the search process (2,668 in the databases search, 31 in the manual search) and sent to screening. 115 papers were selected for inclusion in the literature review (see Appendix 2). These were grouped into the themes of general spirituality, which were discussed in the introduction, and Islam and health, Social etiquette, Cancer screening, Diet, Medications and their alternatives, Ramadan, Hajj, Mental health, Organ donation and transplants, and End of life.

### 3.1 Strengths

• This paper updates a review of the literature on this topic, including papers published as recently as 2021.
• The writing team includes a balanced mixture of Muslim and non-Muslim clinicians with experience in caring for Muslim patients from a variety of disciplines: primary care, hospital care, pharmacy, and mental health. The majority of the team has practiced in both Muslim and non-Muslim majority countries. For further details, please see the “About the Authors” section at the end of the paper.

### 3.2 Limitations

• Due to the relatively low amount of research in investigating the inequities experienced by Muslim patients in non-Muslim majority settings, some of the studies are in subsets of Muslim patients, particularly refugee and immigrant populations. Caution is needed in the interpretation and application of the results of these studies, which is pointed out in the text.

### 3.3 Review of the literature

#### 3.3.1 Islam and health

Islam encourages Muslims to seek treatment when they are unwell[46], [47] while ascribing meaning to illness as a test from God that can expiate, or extinguish guilt from, sin. Prophet Muhammed (PBUH)* said, “No fatigue, nor disease, nor sorrow, nor sadness, nor hurt, nor distress befalls a Muslim, even if it were the prick he receives from a thorn, but that Allah expiates some of his sins for that.”[48] Islamic practice teaches Allah rewards not only the patient suffering from the illness in the hereafter, but also the visitors and family members who care for and express concern for that patient. The combination of these values means that Muslim patients may deal with illness in a variety of ways; they may resort to modern medicine, traditional treatment, or spiritual healing through reading the Noble Qur’an, the holy book for Muslims, or a combination of treatments. Therefore, when taking a patient history, it is essential to ask questions about previous treatments, including complementary and alternative medicine. It is also important to note that traditional treatment and spiritual practices may be additionally influenced by cultural and geographical factors. Care teams should support the patient holistically and may, where appropriate, include the collaboration of pharmacists[49], nurses[50], chaplains[50], [51], [52], case managers[53], and community health workers[54]. Additionally, in neighborhoods with a significant proportion of Muslim members, leaders of Muslim faith communities can be powerful allies in health promotion and prevention.[55], [56], [57].

#### 3.3.2 Social etiquette

Awareness of Islamic social etiquette will aid in the building of clinician-patient rapport and avoid a negative first impression. Lack of awareness can conversely lead to distrust and miscommunication, which are associated with poorer health-care outcomes.[58] The standard Muslim greeting is “Asalaam aleikum,” to which the usual reply is “Wa aleikum asalaam.” A rough English translation is “Peace be on you” and “And peace be upon you.” Muslims come from many different ethnic, cultural, and linguistic backgrounds, but this standard greeting is accepted by all Muslims, and the use of this greeting may endear a patient to those who offer it, helping to build trust and connection.

It is common in many countries and cultures to introduce oneself by shaking hands. However, for Muslim men and women, preserving their modesty and privacy is important[59] and therefore it is considered inappropriate for a man and woman who are unrelated to shake hands. Care teams should be aware of this and allow the patient to decide if their greeting includes a handshake. This can be achieved by being aware of the patient’s cues (i.e allow the patient to extend or withhold their hand first) during the initial interaction.

Likewise, prolonged eye contact can also be uncomfortable, especially between genders. Clinicians need to be aware of this and sensitive to the patient’s preferences. Furthermore, some patients will express a strong preference to be attended by a clinician from the same gender[60], [61], and where this is possible, patient autonomy should be respected. Nevertheless, it is permissible, especially in urgent or emergency situations, for a male clinician to touch a female patient for a physical examination or procedure, and vice versa.[62] Where possible, communication should be clear and consent should be sought. However, exposure of the body should be limited to the area being examined and, once examined, should be covered again promptly.

Family plays a central role in the lives of many Muslims. Clinicians should respect patient autonomy and confidentiality, while appropriately involving family members if preferred by the patient.[63] These preferences can be elicited by asking the patient rather than by making assumptions. If family members are present, they can be asked to sit behind a curtain during any physical examinations.

Some topics, such as alcohol use, smoking, and sexual practices, are inherently sensitive to most people. This is especially true in the case of Muslim patients. It may be helpful to introduce topics of query slowly, explaining how it affects patient care, together with a normalizing phrase such as, “I’m sorry, but I ask all my patients about their habits. Would it be okay if I ask you about […]?” It may benefit the long-term therapeutic relationship to defer some lines of questioning to subsequent visits where possible, rather than pursue them at the initial visit.

In Islam, the left hand is considered ceremonially unclean, so where possible, the right hand should be used when offering medication, food and drink, tissues, and so on.

#### 3.3.3 Cancer screening

One of the aims of primary care is to prevent disease or to detect disease before it negatively affects patients’ health. The benefit of screening is highly dependent on the proportion of the population participating in testing, and lack of understanding and accommodation of Islamic religious requirements may increase reluctance to utilize healthcare[64] and negatively impact preventive care such as colorectal[65], cervical[66], [67], and breast cancer[68] screening rates, contributing to delayed diagnosis and inequitable outcomes. However, this evidence is limited by most of studies being performed in recent Muslim immigrants and may not be generalizable to the greater Muslim community. One study from Chicago, which included a significant number of African American Muslim women along with Arab American and South Asian American Muslim women, did not confirm the effect of modesty concerns on cervical cancer screening, perhaps because it examined both recent immigrants and Muslims who had resided in the area for longer periods of time.[67] Interestingly, all of the included studies found that a relationship with a primary care provider resulted in improved screening rates [65], [66], [67], [68], [69], [70], [71], which supports the role of primary care in cancer screening. The challenges and barriers mentioned previously may be more prevalent for Muslim women. An older survey found that over 93 % of Muslim women in a small sample (n = 27) reported that their clinician did not understand their religious or cultural needs which led to distrust in the healthcare system and missed opportunities for care.[64] Studies have shown that for intimate exams such as mammography[70], clinical breast or pelvic exams[71], [72], a female clinician is strongly preferred. Non-Muslim women often also prefer a female clinician, however, the effect of this on screening rates was higher in Muslim women. One study reported a willingness to participate in self-sampling for HPV screening where appropriate.[73] Given the effectiveness of human papillomavirus (HPV) vaccination in preventing cervical cancer and the mechanism of its transmission, discussion of HPV vaccination requires awareness of cultural and religious beliefs and sensitivity in communication to adolescents and their parents.[74].

#### 3.3.4 Diet

Diet is central to the prevention and treatment of many diseases[75], and as such, a basic understanding of Islamic belief about diet may be helpful while incorporating this into the patient’s personal non-religious preferences. Islam requires Muslims to abstain from a short list of food and beverages. The Arabic word that is used for permissible and non-permissible in Islamic texts is Halal and Haram, respectively.[76] The concept of Haram goes beyond simply avoiding the consumption of alcohol or pork. To be certified as Halal, meat should be taken from animals killed in compliance with halal requirements, as the method of slaughter and subsequent processing determine whether it is acceptable for consumption.[77] This may be more problematic for patients admitted to a hospital setting for inpatient management[78], where family or friends may have to make special efforts to obtain certified halal food that also serves the clinical requirements of the patient, especially if restrictions are in place, such as a renal or low-sodium diet. If the hospital cannot obtain Halal-certified beef, lamb, or poultry, and family or friends cannot supply a suitable meal, then fish, egg-based entrees, and vegetarian food are halal. However, some methods of food handling or production, such as the extraction of vanilla using alcohol or the presence of glycerin from forbidden sources, may result in food being considered objectionable to Muslims.

#### 3.3.5 Medications and their alternatives

Treatment outcomes are directly associated with the percentage of time a patient follows their treatment regimen which is related to their trust in their clinician.[79] As such, cultural sensitivity may guide medication selection and patient counseling, supporting treatment adherence. Some medications may include ingredients that are not permissible to Muslims, such as alcohol, pork, or animal derivatives such as gelatin and heparin. For example, some cough syrups contain alcohol that makes them forbidden. Table 1 shows a list of potentially objectionable medications from animal sources, and Table 2 shows a list of medications containing alcohol. Alternative formulations, such as tablets, gel caps, or alcohol-free solutions, should be substituted when available, and collaboration with pharmacists in the prescribing of medications may avoid inadvertent exposure to undesired medications.

**Table 1:** Medications of animal origin.

Therapeutic class/indication	Pharmacologic category	Generic name	Source	Possible alternatives
Asthma	Interleukin-4 receptor antagonist	Dupilumab	CHO cells†	None
Asthma	Interleukin-5 receptor antagonist	Benralizumab	CHO cells†	None
Asthma	Monoclonal antibody	Omalizumab	CHO cells†	None
Anticoagulant	Factor X inhibitor	Heparin sodium	Porcine	Fondaparinux
Anticoagulant	Factor Xa inhibitor	Dalteparin, Enoxaparin, Tinzaparin	Porcine	Fondaparinux
Anticoagulant	Heparinoid	Danaparoid	Porcine	Fondaparinux
Anticoagulant	Platelet glycoprotein IIb/IIa receptor inhibitor	Abciximab	Murine (mouse)	Tirofiban, Eptifibatide
Antihemophilic agents	Coagulation factors VIII, IX, recombinant factor VIII	Nonacog alfa, Recombinate antihemophilic factor, Moroctocog alfa	CHO cells†	No alternatives
Antihemophilic agents	Coagulation factors VIIa, VIII	Eptacog alfa, Octocog alfa	BHK cells‡	No alternatives
Antihemophilic agents	Prothrombin complex concentrates (PCCs)	Factors II, VII, IX, X, & heparin (porcine)	Porcine	Profilnine (3 factor PCCs)
Antineoplastic	Anti-CD20	Rituximab Obinutuzimab Ofatumumab	CHO cells† (Rituximab, Obinutuzimab), Murine (Ofatumumab)	Ibrutinib
Antineoplastic	Anti-CD52	Alemtuzumab	CHO cells†	None
Antineoplastic	Anti-HER2	Trastuzumab Pertuzumab	CHO cells†	Lapatinib Neratinib
Antineoplastic	Anti-PD-1 monoclonal antibody	Pembrolizumab	CHO cells†	None
Antineoplastic	EGFR inhibitor	Panitumumab Cetuximab	CHO cells† (Panitumumab) Chimeric (Cetuximab)	Regorafenib
Antineoplastic	Monoclonal antibody	Daratumumab	CHO cells†	Bortezomib
Antineoplastic	VEGF inhibitors	Bevacizumab Ranibizumab Ramucirumab	Murine (mouse)	Regorafenib
Antirheumatoid	Tumor necrosis factors	Infliximab Adalimumab Golimumab	Various	Tofacitinib Baricitinib
Antivenom	Immune globulin antivenin	Snake type antivenom	Equine	None
Complementary osteoarthritis	Anabolic/anti-inflammatory	Chondroitin	Porcine/bovine/avian/shark/fish	Halal chondroitin source
Disease modifying	Interferon	Interferon beta-1a	CHO cells†	Interferon beta-1b
Disease modifying	Tumor necrosis factors	Abatacept Certolizumab Etanercept	Various animals	None
Enzyme	Enzyme replacement	Laronidase Galsulfase	CHO cells†	None
Enzyme	Enzyme replacement	Imiglucerase	CHO cells†	Velaglucerase alfa
Enzyme	Enzyme replacement	Agalsidase beta	CHO cells†	Agalsidase alfa
Fibrinolytic agent	Thrombolytic	Tenecteplase Alteplase	CHO cells†	Reteplase
Hormone	Estrogen	Conjugated estrogens	Equine	Estratab Estrace
Gonadotropin	Ovulation stimulator	Follitropin Corifollitropin	CHO cells†	Urofollitropin, menotropins
Gonadotropin	Ovulation stimulator	Choriogonadotropin alfa	CHO cells†	Human chorionic gonadotropin
Growth hormone	rHGH§	Somatropin	Murine (mouse)	Norditropin Humatrope Omnitrope
Hemopoietic	Colony-stimulating factor	Epoetin Darbepoietin	CHO cells†	None
GI	Digestive enzymes	Betaine Pepsin	Porcine	Halal bovine source
Immunoprophylactic	Monoclonal antibody	Palivizumab	Murine (mouse)	None
Immunosuppressive agent	Interleukin-2 inhibitor	Basiliximab Daclizumab	Murine (mouse)	None
Osteoporosis	Monoclonal antibody	Denosumab	CHO cells†	Bisphosphonates
Pancreatic enzyme	Pancrelipase	Amylase, lipase, Pancrelipase, Protease	Porcine	None
Respiratory	Lung surfactant	Poractant alfa	Porcine	Calfactant Beractant
Hemopoietic	Colony-stimulating factor	Lenograstim	CHO cells	Filgrastim Pegfilgrastim

†Chinese hamster ovary cells.

‡Baby hamster kidney cells.

§Recombinant human growth hormone.

**Table 2:** Commonly used medications containing ethanol with ethanol-free alternatives.

Medication class	Medication	Alternative
*Antidiarrheal*		
	Lomotil liquid 15 %	
*Laxatives*		
	Cascara	
	Senecot syrup	
*Antiemetic*		
	Dramamine liquid	
	Metoclopramide syrup	
	Promethazine syrup	
*Anti-infective*	Bactrim (TMP/SMX) syrup	Depends on indication
*Cough/cold combinations*		
	Various (anti-Tuss DM, Cheracol, Dayquil, Dimetapp, Formula 44, Robitussin, Sudafed, Vicks)	Delsym, Actifed, Triaminic, Tussionex
*Corticosteroids*		
	Dexamethasone	
	Prednisone	
	Prednisolone	
*Supplements*		
*Iron*	Feosol elixir, Fer-in-Sol, Fumeral, Niferix)	
*Multivitamins*	(Geritol, Geraplex)	
*Toothache/cold sore*		
	Anbesol, Betadine mouthwash	Ora-Base with benzocaine, Carmex

In 1995, the World Health Organization and some of the religious leaders of the Islamic faith, including Egypt’s well-respected and widely followed Al Azhar University, hosted a conference that concluded that the use of animal gelatin, even from forbidden animals, is permissible for use in the manufacture of medications and vitamin preparations.[80] However, some Muslims do not accept this conclusion.

One of the central principles of Islamic law is that “necessities overrule prohibitions.”[81] Muslims are permitted to use medications derived from otherwise forbidden sources if no appropriate alternatives are available and the therapeutic indication is urgent.

### 3.5 Ramadan

A particularly challenging time in the treatment of certain Muslim patients is the month of Ramadan, which commemorates the receiving of the first revelation from God to the prophet Mohammed (PBUH). Fasting during this month is one of the five central practices of Islam. Because the Islamic year is based on the lunar calendar of 355 days, Ramadan moves forward by approximately 10 days each year on the Gregorian calendar. During Ramadan, Muslims who are healthy enough to fast are required to abstain from food and drink from shortly before sun up to shortly after sundown. Those who are pregnant or are at extremes of age or have diabetes with an IDF-DAR (International Diabetes Federation- Diabetes and Ramadan Alliance) risk category of high or very high should be exempted from fasting [82]. Because of the variance in the length of day by season and latitude, this can result in varied fasting hours, which, at extremes of latitudes, can result in fasts of up to 20 hours per day or more. Sleeping patterns are also affected during Ramadan[83], because of the performance of additional prayers during the night. These changes may lead to a higher utilization of healthcare resources; one US health system reported a higher emergency department utilization among Muslims compared to non-Muslims during Ramadan, which may indicate an increased rate of acute problems.[84].

Patient-centered planning that is informed by a basic understanding of the Islamic practice of fasting can help patients with chronic conditions to achieve the requirements of the fast with minimal adverse impact on their health.[85] Those who are ill, pregnant, or menstruating are excused from fasting and can make up for missed days of fasting at a later date. Enlisting the help of an Islamic hospital chaplain or other leader, preferably one who is formally trained from a recognized Islamic institution, may lend assurance and credibility when fasting is not medically advisable. The Islamic Society of North America (www.isna.net) may be a useful resource in this process. Adequate pre-fast hydration is essential, especially in situations where the risk of heat-related illness or dehydration is higher, such as in warmer climates or in occupations where physical labor results in increased fluid loss.

Because of the risk of hypoglycemia, insulin is an obvious medication that can be affected by fasting. As discussed earlier, the preservation of life is highly valued in Islam, and patients should be encouraged to break their fast if they experience significant hypoglycemia. It may be beneficial to review rules with patients, such as the 15:15 rule, to ensure that they can self-manage if hypoglycemia occurs, as one study reported 33 % of patients persisted in fasting despite self-identification of hypoglycemia[86]. Recent guidelines advocate for the prescribing of glucagon to those at risk of hypoglycemia, which is available in both injectable and intranasal forms.[87] Adjustment of insulin dosage and timing is often required during the fast. Expert guidelines recommend a consideration of reduction in the total insulin dose by 20 %–50 % in fasting patients.[88] In patients on basal-bolus insulin, mealtime short-acting insulin doses which are normally given during the day when not fasting can be moved to the evening hours to cover for carbohydrate intake at that time.[89] In some cases, significant amounts of carbohydrates are consumed during the evening and night hours[90], resulting in hyperglycemia[91], which may require correction dose insulin or, in patients on a premixed insulin regimen, a change in the mixture from 70/30 to 50/50 for the evening dose[92]. Clear and open communication between clinicians and patients as to the patient’s care plan during Ramadan is essential to avoid excessive fluctuations in blood glucose levels[92], [93], but may be hampered by knowledge^,^[94], [95], [96], [97] and cross-cultural gaps[98], as well as limited visit times and competing clinical priorities.[99].

Given the prohibition of oral intake during fasting hours, oral medications that require multiple daily doses may be affected. Acetaminophen and ibuprofen, for example, are commonly dosed every 6 h for pain management. A medication review may be required with shared decision-making regarding the use of alternative medications, such as naproxen or celecoxib, if clinically appropriate, which can be taken less frequently.[100].

Another clinically significant consideration are other routes by which medications are given during fasting hours. Some Islamic scholars have ruled that, except for intravenous fluids or feeding, medications, as well as injections, administered to the eyes, ears, nose, and skin are permitted.[101] Muslims who follow stricter interpretations of Islam may refuse medications administered via some other routes, including nasal sprays[102], because of concerns about invalidating their fast due to the possibility of inadvertently swallowing material that may enter the throat. Patients may also refuse rectal or vaginal suppositories during fasting hours as well. Topical treatments are generally acceptable, and there has been success with the use of topical NSAIDs for analgesia where appropriate.

Smoking is also prohibited during the daytime hours of Ramadan, which may represent an opportunity to set a quit date for those interested in smoking cessation[103].

### 3.6 Prayer

Muslims practice prayer five times a day: before dawn, at midday, at midafternoon, at sundown, and at night. Ritual cleansing is performed prior to prayers. This is typically done with water, but in cases where water is unavailable, alternative ways to prepare for prayer are available. The offering of prayers does not require a great deal of time, so where possible, it is often appreciated that Muslims are excused while they pray, and whatever was happening prior to the time of prayer can be resumed afterward.

### 3.7 Hajj

Another important component of Islamic life is performing pilgrimage, or Hajj, once in a lifetime. This pilgrimage occurs once a year, and typically over a million Muslims from around the world gather to perform the pilgrimage. As pilgrims gather to perform rites, they experience crowded conditions for the few days around and during Hajj, increasing the risk of acquiring communicable diseases[104], [105], [106]. Pretravel counseling should include infection prevention measures such as vaccination, the use of face masks, and appropriate hand and food hygiene.[107], [108], [109] Saudi Arabia, where Hajj occurs, currently requires non-Saudi visitors to provide a negative COVID-19 PCR 72 h prior to boarding for all travelers above the age of 8, and a valid health insurance policy that includes care for COVID-19 is required. Non-Saudis who are not vaccinated against COVID-19 must quarantine for seven days in a hotel or shelter approved by the Saudi ministry of tourism; vaccinated travelers (pre-travel registration is required) are not required to quarantine, but not all WHO-approved vaccines are accepted for fulfillment of this criteria.[110] At the time of writing, Saudi Arabia requires that the quadrivalent meningococcal vaccine must be documented as given at least 10 days prior and no more than 3 years before arrival in the country. For pilgrims transiting through other countries, other vaccines such as yellow fever and polio, may be required. Influenza vaccination is recommended. As pilgrims return to their home countries, clinicians should be alert to this portion of their travel history[111] in the event of an outbreak of an infectious disease. The website https://www.cdc.gov is a reliable source of information regarding ongoing outbreaks of infectious diseases around the world.

### 3.8 Mental health

Despite the increase in mental health challenges that Muslims face these days, there is general hesitation in seeking professional mental health care.[112] Arab-Americans are less likely to be screened for depression, and are similarly less likely to follow up with a behavioral health specialist, but they are more likely to follow up with a primary care clinician[113]. Muslim immigrants specifically may face a different set of issues, including a lack of understanding of Western healthcare systems, misunderstanding of terms used to describe mental health symptoms and diagnoses, and certain beliefs or perceptions of healthcare treatment [114]. In a paper studying older Somali immigrants in Finland, the participants indicated a preference for informal healthcare over conventional healthcare and were more likely to consult religious figures or general practitioners/primary care providers than mental health professionals, highlighting the important role of general practitioners in the wellness of Muslim patients[115]. This could be due to the belief that a therapist will not provide religion-based intervention.[116], [117] Research suggests that the incorporation of religious practices in secular healthcare frameworks is generally beneficial in patients that consider themselves highly religious[118], [119]. The use of more informal or religious healthcare practices in conjunction with conventional medicine could contribute to more culturally sensitive care— with regards to Muslim patients specifically, participation in Quran readings, Islamic therapy, and community counseling[114], [120]. It is possible that therapeutic frameworks that do not integrate religious concepts may further alienate Muslims seeking care in a Western healthcare system, illustrating how crucial an Islamic-oriented approach can be.[112] Furthermore, awareness of the stigmas surrounding mental health, and attempts to provide culturally relevant care, especially by primary care providers (such as same-gender clinicians, halal healthcare plans, and Islamic mental health treatment), could reduce some of the inequities faced by Muslim patients[114], [120], [121].

### 3.9 Organ donation and transplants

Muslim patients may require transplants or may be interested in organ donation. Most Muslims find this acceptable whereas others do not. The Fiqh Council of North America agreed with many other international Islamic councils that organ donation and transplantation are permissible within the Islamic faith and among American Muslims, with several caveats. The ruling considers organ donation to be a rewarded act of charity and generally aligns with mainstream standards of practice in prohibiting the selling of organs, requiring donor consent, and the need for a medical team to assess possible harm to the donor. The ruling explicitly prohibits the donation of reproductive organs such as ova, sperm, and the uterus.[122] Where it differs from most current practice is that it historically has required donation of a “vital organ” to occur after cardiac determination of death. This may limit the types of organs that can be donated, especially in cases of heart transplantation. However, since 1986 in Saudi Arabia, organ transplantation has been allowed from persons after brain death, and, by the end of 2008, more than 3,600 organs had been transplanted in cases meeting these criteria.[123] The determination of death and other end-of-life issues will be discussed further, below.

### 3.10 Childbirth and breastfeeding

It is customary for a Muslim father to whisper a prayer into his newborn’s ears immediately after birth. If the baby’s clinical condition permits, this should be allowed to respect this religious practice. Some Muslims also place a date or honey into the baby’s mouth, and clinicians providing delivery care to Muslim patients may want to discuss this as part of the birth plan, as honey presents a potential botulism risk[124], [125], [126].

Mothers are highly encouraged to breastfeed for 2 years, which aligns with current clinical recommendations and guidelines.[127].

### 3.11 End of life

With regard to worldview as it pertains to health issues, the Islamic faith teaches that God holds a position of sovereignty over life.[128] This is balanced by personal and societal responsibility to care for one’s health as a gift that has been given to us. The visibility of these principles, which may be in tension, is often magnified at the end of life. End-of-life care does not simply require dealing with physical suffering. It also requires supporting patients who are coming to a place of closure.

#### 3.11.1 Advance directives

End-of-life issues can be fraught with mixed emotions. Research has found that the family members of those patients who die without an advanced directive in place are more likely to report concerns regarding physician communication and anticipatory guidance.[129] Non-Muslim clinicians may be less likely to offer advance care planning to Muslims even though completing advanced directives leads to better communication and more informed, realistic expectations among family members.[130], [131].

Islam values the relief of suffering, but, as with many other cultures and religions, it is forbidden to actively hasten death.[132] As such, the completion of written advance directives such as do-not-resuscitate (DNR) orders, or documents such as California’s Physician Orders for Life Sustaining Treatment, require careful listening to and understanding of the patient’s and family’s wishes. The patient may require guidance from clinicians throughout several interactions[133] to contemplate what their wishes are. These sessions are best performed in a location free of distraction and time pressures. Medical jargon should be avoided, and explanations of what certain terms mean should be explained in a manner appropriate to the patient, and with consent, the patient’s family[134], taking into account such factors as language, culture, educational level, and health literacy[135], [136].

#### 3.11.2 Definition of death

Many Islamic scholars and medical organizations have recommended that brain death be considered clinical death, but this remains an area of controversy. Some Muslim clinicians, Muslim patients, and their families may continue to use cardiac death as the definition of death.[137] This has ramifications for organ donation[138] and clinical decisions regarding medical futility.[139] Nonjudgmental listening and communication, enlisting the assistance of an Islamic leader[128], time-limited trials of therapy,[140] and consultation with a hospital ethics committee may be beneficial in arriving at a mutually agreeable care plan.

#### 3.11.3 Burial practices

Islamic law requires the burial of the body as soon as possible after death. A few rituals are enacted immediately prior to and immediately after death. The body should then be covered completely, including the face, by a clean sheet. Those of a different gender to the deceased should avoid handling the body as much as possible. Islamic law prohibits the disfigurement of the body after death, so autopsy requests may be met with resistance. However, in cases where autopsy is medicolegally necessary, with appropriate communication, agreement may be reached. The body is usually ritually washed and prepared for burial by relatives or other members of the Muslim community, according to Islamic requirements.[141] A full list and explanation of procedures are beyond the scope of this article, but a helpful resource is provided in Table 3 [insert Table 3].

**Table 3:** Additional resources for caring for Muslim patients:

Cultural Competence in the Care of Muslim Patients and Their Families	https://www.ncbi.nlm.nih.gov › books › NBK499933
Queensland Health and Islamic Council of Queensland. Health Care Providers’ Handbook on Muslim Patients Second Edition 2010	https://www.health.qld.gov.au/__data/assets/pdf_file/0034/155887/islamgde2ed.pdf
Irish Hospice Foundation	https://hospicefoundation.ie/wp-content/uploads/2013/04/Caring-for-A-Muslim-Patient.pdf

## 4 Conclusion

Effective, equitable medical care in the modern era requires the integration of scientific, quantitative, evidence-based measures of quality with the unquantifiable elements of holistic patient-centered care. This requires both knowledge of the disease process and an understanding of the patient as an individual, regarding how they think and feel and are integrated into their family and community. Medical knowledge in isolation is insufficient to deliver patient-centered care and achieve optimal outcomes. As the number of Muslim–Americans increases, a basic knowledge of Islamic practices and beliefs as they pertain to clinical care has the potential to avoid diagnostic errors, support medical decision-making, and strengthen the clinician–patient relationship to great therapeutic effect. Because of the limited amount of research to date on Islamic practice and its effects on the practice of medicine, further research should be encouraged and funded to direct specific practices to reduce inequities in the healthcare of Muslim patients.

The views expressed in this presentation are those of the authors and do not necessarily reflect the position or policy of the Department of Veterans Affairs or the US government.

## Funding

Authors created this manuscript as part of their employment or affiliation with their organization. No grants or other external funding were involved.

The authors would like to thank Reem AlSheryani and Mariam AlAhbabi from the National Medical Library for their tireless work in obtaining papers for review.

*PBUH: May peace be upon him. This is usually included after a mention of any of the Prophet Mohammed’s names as a sign of respect.

About the Authors.

The writing team was comprised of authors from several different disciplines.

Primary care:

Dr. Jeffrey King, a Christian, cared for Muslim patients for 6 years in a government primary health care center and taught in the clinical setting in the UAE for 9 years with students, interns and residents.

Dr. Alexander Kieu, also a Christian, is trained and board certified in the US. He practiced for 7 years in the US and is currently caring for Muslim patients at Kanad Hospital Al Ain for the past three and a half years, teaching in the clinical setting with Emirati medical students.

Professor Muhammad Jawad Hashim, a Muslim, is a professor of family medicine teaching in the preclinical and clinical setting with medical students and residents in the UAE. He was trained and is board certified in the US with over 20 years of clinical experience.

Dr. Moien AB Khan, a Muslim, is the chair of the Family Medicine Department at UAEU, with 20 years of clinical experience in caring for patients and teaching students, interns, and residents in the UK and the UAE.

Dr. Romona Devi Govender, an agnostic, has 34 years of clinical experience in caring for patients in South Africa, of which 20 years have been spent teaching students, interns, and residents in clinical settings in South Africa and the UAE.

Pharmacy:

Dr. El-Deyarbi, a clinical pharmacist, has 20 years of experience in caring for Muslim patients in the outpatient and inpatient settings, and has added both his own personal understanding and practice of Islam as well as generating the table of medications that require consideration when used in Muslim patients.

Mental health:

Dr. Renee King, Ms. Aljneibi, and Ms. King reviewed and analyzed the identified papers and collaborated on the writing of this section. Dr. Renee King, a Christian, has six years of clinical experience and nine years of living in the UAE, a Muslim majority Arab country. Ms. Aljneibi, a Muslim, was director of the Emirates Center for Happiness Research, having completed her MA in Positive Psychology at the University of Pennsylvania, which has given her insight into the care of both Muslim and non-Muslim patients.

Hospital care:

Dr. Saif Al Shamsi, a Muslim, completed his training at Dalhousie University in Canada, and has 10 years of experience in caring for inpatients at Tawam Hospital in the UAE, as well as 13 years of clinical teaching Emirati students, interns, and residents, which has given him insight into the care of both Muslim and non-Muslim patients..

Medical library information science:

Ms. Östlundh has 25 years of multicultural library leadership and research support experience, of which she spent 3 years at Weill-Cornell Medicine-Qatar and 9 years at Zayed University and the College of Medicine and Health Sciences at United Arab Emirates University. She has additional experience from living in and working on a library related project in Egypt. Ms. Östlundh devised the thorough literature search performed at the outset of our project and has contributed her own personal insight as a non-Muslim living in three Muslim majority Arab countries to the writing of our manuscript.

## Declaration of Competing Interest

The authors declare that they have no known competing financial interests or personal relationships that could have appeared to influence the work reported in this paper.

## Figures and Tables

**Table:** 

**Source and date**	**Search strategy**	**Results**	**Notes**
**PubMed** (NLM) **Search date:** 2022–01-28	((“mohammedan*” OR “muslim*” OR “Islam*” OR “religion*”[Title/Abstract] OR “religious”[Title/Abstract] OR “Religion and Medicine”[MeSH Terms] OR “Religion”[Mesh:NoExp]) AND (“primary healthcare”[Title/Abstract] OR “primary health care”[Title/Abstract] OR “primary health care”[Title/Abstract] OR “primary health care”[MeSH] OR “primary care”[Title/Abstract] OR “primary medical care”[Title/Abstract] OR “family medicine”[Title/Abstract] OR “Family Practice”[MeSH Terms] OR “family practice”[Title/Abstract] OR “family physician*”[Title/Abstract] OR “family practitioner*”[Title/Abstract] OR “family clinic*”[Title/Abstract] OR “general practice”[Title/Abstract] OR “general practitioner*”[Title/Abstract] OR “General Practitioners”[MeSH] OR “general clinic*”[Title/Abstract] OR “general physician*”[Title/Abstract] OR “ambulatory care”[Title/Abstract] OR “ambulatory health-care”[Title/Abstract] OR “ambulatory healthcare”[Title/Abstract] OR “ambulatory health-care”[Title/Abstract] OR “ambulatory clinic*”[Title/Abstract]OR “ambulatory physician*”[Title/Abstract] OR “ambulatory practitioner*”[Title/Abstract] OR “ambulatory health center*”[Title/Abstract] OR “Ambulatory Care Facilities”[MeSH]))	**2,201**	All search terms are searched in the fields: “title” and “abstract” (here marked with “TI/AB”) and in “MeSH” when available. Filters for English Language and publication year 1995-search date is applied.
**Scopus** (Elsevier) **Search date:** 2022–01-28	((TITLE-ABS(“primary healthcare” OR “primary health care” OR “primary health-care” OR “primary care” OR “primary medical care” OR “family medicine” OR “family practice” OR “family physician*” OR “family practitioner*” OR “family clinic*” OR “general practice” OR “general practitioner*” OR “general clinic*” OR “general physician*” OR “ambulatory care” OR “ambulatory health care” OR “ambulatory healthcare” OR “ambulatory health-care” OR “ambulatory clinic*” OR “ambulatory physician*” OR “ambulatory practitioner*” OR “ambulatory health center*”) AND TITLE-ABS(mohammedan* OR “muslim*” OR “Islam*” OR “religion*” OR “religious”))	**1,025**	All search terms are searched in the fields: “title” and “abstract” (here marked with “TITLE-ABS”). No MeSH/Subject Headings available in Scopus. Filters for English Language and publication year 1995-search date is applied. 1995 and Eng only
**CINAHL** (EBSCOhost) **Search date:** 2022–01-28	((TI (“primary healthcare” OR “primary health care” OR “primary health-care” OR “primary care” OR “primary medical care” OR “family medicine” OR “family practice” OR “family physician*” OR “family practitioner*” OR “family clinic*” OR “general practice” OR “general practitioner*” OR “general clinic*” OR “general physician*” OR “ambulatory care” OR “ambulatory health care” OR “ambulatory healthcare” OR “ambulatory health-care” OR “ambulatory clinic*” OR “ambulatory physician*” OR “ambulatory practitioner*” OR “ambulatory health center*”) OR AB (“primary healthcare” OR “primary health care” OR “primary health-care” OR “primary care” OR “primary medical care” OR “family medicine” OR “family practice” OR “family physician*” OR “family practitioner*” OR “family clinic*” OR “general practice” OR “general practitioner*” OR “general clinic*” OR “general physician*” OR “ambulatory care” OR “ambulatory health care” OR “ambulatory healthcare” OR “ambulatory health-care” OR “ambulatory clinic*” OR “ambulatory physician*” OR “ambulatory practitioner*” OR “ambulatory health center*”) OR (MH “Family Practice” OR “Physicians, Family” OR “Ambulatory Care Facilities+” OR “Primary Health Care”) AND (TI (mohammedan* OR “muslim*” OR “Islam*” OR “religion*” OR “religious”) OR AB (mohammedan* OR “muslim*” OR “Islam*” OR “religion*” OR “religious”) OR (MH “Islam” OR “Religion and Religions” OR “Religion and Medicine”))	**670**	All search terms are searched in the fields: “title” and “abstract” (here marked with “TI” and “AB”) and in CINAHL’s Subject Headings (here marked with “MH”) when available). Filters for English Language and publication year 1995-search date is applied.
**Total numbers of records identified:**		**3,896**	
**Total numbers of unique records identified after de-duplication:**		**2,668**	

## Appendix

### Appendix 1: Literature search strategies

#### Literature search

Appendix 2.

**Included papers** (Reference number in the text precedes each reference in parentheses).

1. (7) Sager E. Components of successful spiritual care. *J Religion and Health.* 2020; 1–16. https://doi.org/Doi.org/10.1007/s10943-020–01089-2.
2. (10) Isaac K, Hay J, Lubetkin E. Incorporating spirituality in primary care. *J Religion and Health.* 2016; 55(3):1065–1077. DOI https://doi.org/10.1007/s10943-016–0190-2.
3. (11) Waldfogel S. Spirituality in medicine. *Primary Care: Clinics in Office Practice.* 1997; 24(4): 963–976.
4. (12) Winslow GR, Wehtje-Winslow BJ. Ethical boundaries of spiritual care. *Med J Australia.* 2007; 186:S63-66.
5. (13) Kliewer S. Allowing spirituality into the healing process*. The Journal of Family Practice*. 2004; (53):8.
6. (14) MacLean CD, Susi B, Phifer N, et al. Patient preference for physician discussion and practice of spirituality. *J Gen Int Med.* 2003; 18(1):38–43.
7. (15) McCord G, Gilchrist VJ, Grossman SD, et al. Discussing spirituality with patients: A rational and ethical approach. *Annals of Family Medicine.* 2004; 2(4):356–361.
8. (16) Puchalski CM. Formal and informal spiritual assessment. *Asian Pacific Journal of Cancer Prevention.* 2010; 11, MECC Supplement, 51–57.
9. (17) Maddox M. Spiritual assessments in primary care. *The Nurse Practitioner.* 2002; 27(2): 12–14.
10. (18) Curlin FA, Roach CJ. By intuitions differently formed: How physicians assess and respond to spiritual issues in the clinical encounter. *The American Journal of Bioethics.* 2007; 7.7:19–20.
11. (20) D’Souza R. The importance of spirituality in medicine and its application to clinical practice. *Med J Australia.* 2007; 186: S57-59.
12. (21) Kuczewski MG. Talking about spirituality in the clinical setting: Can being professional require being personal? *The American Journal of Bioethics*. 2007; 7(7):4–11. https://doi.org/10.1080/15265160701399545.
13. (22) Hamilton IJ, Morrison J, Macdonald S. Should GPs provide spiritual care? *Br J Gen Pr.* 2017. DOI https://doi.org/10.339/bjgp17X693845.
14. (23) US Department of Health and Human Services. Think cultural health: A physician’s practical guide to culturally competent care. https://cccm.thinkculturalhealth.hhs.gov/. Accessed October 19, 2021.
15. (25) Boulet JR, Duvivier RJ, Pinsky WW. Prevalence of International Medical Graduates from Muslim-majority nations in the US Physician Workforce from 2009 to 2019. *Jama Network Open.* 2020; 3(7)e209418. https://doi.org/10.1001/jamanetworkopen.02020.9418.
16. (26) Rothschild SK. Cross-cultural issues in primary care medicine. *Dis Mon.* 1998; 44(7): 293–319.
17. (27) Vermandere M, De Lepeleire J, Smeets L, et al. Spirituality in general practice: a qualitative evidence synthesis. *Br J Gen Pr.* 2011. DOI: 10.0.99/bjgp11X606663.
18. (28) Meeuwesen L, Harmsen JAM, Bernsen RMD, et al. Do Dutch doctors communicate differently with immigrant patients than with Dutch patients? *Social Science and Medicine.* 2006; 63:2407–2417. https://doi.org/10.1016/j.socscimed.2006.06.005.
19. (29) Odeh Yosef, AR. Health beliefs, practice, and priorities for health care of Arab Muslims in the United States: Implications for nursing care. *Journal of Transcultural Nursing.* 2008; 19(3):284–291.
20. (30) Alzubaidi H, McNamara K, Browning C, et al. Barriers and enablers to healthcare access and use among Arabic-speaking and Caucasian English-speaking patients with type 2 diabetes mellitus: a qualitative comparative study. *BMJ Open.* 2015; 5:e008687. https://doi.org/10.1136/bmjopen-2015–008687.
21. (31) Padela AI, Gunter K, Killawi A, Heisler M. Religious values and healthcare accommodations: Voices from the American Muslim community. *J Gen Int Med*. 2012; (27), 708–715.
22. (32) Padela, AI, Zaidi D. The Islamic tradition and health inequities: A preliminary conceptual model based on a systematic literature review of Muslim health-care disparities. *Avicenna J Med*. 2018; Jan-Mar, 8(1):1–13.
23. (33) Laird LD, Amer MM, Barnett ED, Barnes LL. Muslim patients and health disparities in the UK and the US. *Arch Dis Child.* 2007; (92):922–926. https://doi.org/10.1136/adc.2006.104364.
24. (34) Martin, MB. Perceived discrimination of Muslims in health care. *J Muslim Mental Health*. 2015; 9(2). https://doi.org/10.3998/jmmh.10381607.0009.203
25. (35) Chaouni SB, Smetcoren A, De Donder L. Caring for migrant older Moroccans with dementia in Belgium as a complex and dynamic transnational network of informal and professional care: A qualitative study. *International Journal of Nursing Studies*. 2020; 101:103413. https://doi.org/10.1016/j.ijnurstu.2019.103413.
26. (36) Ashai SA. The Muslim Community Center Clinic: A Maryland safety net clinic, achievements and challenges. *The Journal of Islamic Medical Association of North America.* 2010; 42.3:117
27. (37) Samuels EA, Orr L, White EB, et al. Health care utilization before and after the “Muslim Ban” executive order among people born in Muslim-majority countries and living in the US. *JAMA Network Open.* 2021; 4(7):e2118216. DOI https://doi.org/10.1001/jamanetworkopen.2021.18216.
28. (39) Padela AI, Gunter K, Killawi A, Institute for Social Policy and Understanding. Meeting the healthcare needs of American Muslims: Challenges and strategies for healthcare settings. 2011.
29. (40) Orr RD. Incorporating spirituality into patient care. *AMA Journal of Ethics.* 2015; 17(5):409–415.
30. (46) Al Shahri MZ, Al Khenaizan A. Palliative care for Muslim patients. *Journal of Supportive Oncology.* 2005; 3:432–436.
31. (47) Sullivan L, ed. *Healing and Restoring: Health and medicine in the world's religious traditions.* New York: MacMillan Publishing Company; 1989:149–172.
32. (49) Almansour HA, Chaar B, Saini B. Fasting, diabetes, and optimizing health outcomes for Ramadan observers: A literature review. *Diabetes Therapeutics.* 2017; 8:227–249. DOI https://doi.org/10.1007/s13300-017–0233-z.
33. (50) Donesky D, Sprague E, Joseph D. A new perspective on spiritual care: Collaborative chaplaincy and nursing practice. *Advances in Nursing Science.* 2020; 43(2): 147–158. https://doi.org/10.1097/ANS.0000000000000000298.
34. (51) De Vries R, Berlinger N, Cadge W. Lost in translation: The chaplain’s role in health care. Hastings Center Report. 2008. 38(6):23–27.
35. (52) Harris S. Chaplains’ roles as mediators in critical clinical decisions. *AMA J Ethics.* 2018; 20:E670-674. https://doi.org/10.1001/amaethics.2018.670.
36. (53) LeDoux J, Mann C, Demoratz M, et al. Addressing spiritual and religious influences in care delivery. *Professional Care Management.* 2019; 24(3):142–147. https://doi.org/10.1097/NCM.0000000000000346.
37. (54) Islam N, Nadkarni SK, Zahn D, et al. Integrating community health workers within the Patient Protection and Affordable Care Act implementation*. Journal of Public Health Management Practice.* 2015; 21(1):42–50. https://doi.org/10.1097/PHH.0000000000000084.
38. (55) Chaudhary A, Dosto N, Hill R, et al. Community intervention for Syrian refugees in Baltimore City: The Lay Health Educator Program at a local mosque. *J Religion and Health.* 2019; 58:1687–1697. https://doi.org/10.1007/s10943-019–00893-9.
39. (56) Abu-Ras W, Gheith A, Cournos F. The imam’s role in mental health promotion: A study at 22 mosques in New York City’s Muslim community*. J Muslim Mental Health.* 2008; 3(2):155–176.
40. (57) Padela AI, Killawi A, Heisler M, Demonner S, Fetters MD. The role of imams in American Muslim health: Perspectives of Muslim community leaders in Southeast Michigan*. J Religion and Health.* 2011; 50:359–373.
41. (58) Brach C, Fraserirector I, for the Agency for Healthcare Research and Quality. Can cultural competency reduce racial and ethnic health disparities? A review and conceptual model. *Medical care research and review*. 2000; 57(4 suppl):181–217.
42. (59) Penney D. Listening to Muslim women with refugee backgrounds in the health encounter. J Midwifery & Women’s Health. 2019; 64.5:677. https://doi.org/10.1111/jmwh.13049.
43. (60) Turin TC, Rashid R, Ferdous M, et al. Perceived barriers and primary care access experiences among immigrant Bangladeshi men in Canada. *Fam Med and Comm Health.* 2020; 8:e000453. https://doi.org/10.1136/fmch-2020–000453.
44. (61) Ahmed S, Shommu NS, Rumana N, et al. Barriers to access of primary healthcare by immigrant populations in Canada: A literature review. *J Immigrant Minority Health* 2016; 18:1522–1540. DOI https://doi.org/10.1007/s10903-015–0276-z.
45. (62) Alqufly AE, AlHarbi BM, Alhatlany KK, et al. Muslim female gender preference in delaying the medical care at emergency department in Qassim Region, Saudi Arabia. *J Fam Med and Prim Care.* 2019; 8:1658–63. https://doi.org/10.4103/jfmpc.jfmpc_141_19.
46. (63) Omole FS, Sow CM, Fresh E, et al. Interacting with patients’ family members during the office visit. *American Family Physician*. 2011; 84(7):780–784.
47. (64) Hasnain M, Connell KJ, Menon U, et al. Patient-centered care for Muslim women: Provider and patient perspectives. *J Women’s Health.* 2011; 20(1):73–83. https://doi.org/10.1089/jwh.2010.2197.
48. (65) Vahabi M, Lofters A, Wong J, et al. Fecal occult blood test screening uptake among immigrants from Muslim majority countries: A retrospective cohort study in Ontario, Canada. *Cancer Medicine*. 2019; 8:7108–7122. DOI https://doi.org/10.1002/cam4.2541.
49. (66) Lofters AK, Vahabi M, Kim E, et al. Cervical cancer screening among women from Muslim-majority countries in Ontario, Canada. *Cancer Epidemiology, Biomarkers & Prevention.* 2017; 26(10):1493–1499. https://doi.org/10.1158/1055–9965.*EPI*-17–0323.
50. (67) Padela AI, Peek M, Johnson-Agbakwu CE, et al. Associations between religion-related factors and cervical cancer screening among Muslims in Greater Chicago. *Journal of Lower Genital Tract Disease.* 2014; 18(4):326–332. https://doi.org/10.1097/LGT.00000000000000026.
51. (68) Hasnain M, Menon U, Ferrans CE, et al. Breast cancer screening practices among first generation immigrant Muslim women. *Journal of Women’s Health.* 2014; 23(7):602–612. https://doi.org/10.1089/jwh.2013.4569.
52. (69) Dressler J, Johnsen AT, Madsen LJ, et al. Factors affecting patient adherence to publicly funded colorectal cancer screening programmes: a systematic review. *Public Health.* 2021; 190:67–74. https://doi.org/10.1016/j.puhe.2020.10.025.
53. (70) Bhargava S, Hofvind S, Moen K. Gender, letters, relatives, and God: Mediating actors in mammographic screening among Pakistani women in Norway. *Acta Radiologica Open.* 2019; 8(9):1–15. https://doi.org/10.1177/2058460119875015.
54. (71) George P, Terrion JL, Ahmed R. Reproductive health behaviour of Muslim immigrant women in Canada. *International Journal of Migration, Health and Social Care*. 2014; 10(2):88–101. https://doi.org/10.1108/IJMHSC-09–2013-0032.
55. (72) Vahabi M, Lofters A, Kim E, et al. Breast cancer screening utilization among women from Muslim majority countries in Ontario, Canada. *Preventive Medicine.* 2017; 105:176–183. https://doi.org/10.1016/j.ypmed.2017.09.008.
56. (73) Vahabi M, Lofters A. Muslim immigrant women’s views on cervical cancer screening and HPV self-sampling in Ontario, Canada. *BMC Public Health.* 2016; 16:868. https://doi.org/10.1186/s12889-016–3564-1,
57. (74) Netfa F, King C, Davies C, et al. Knowledge, attitudes, and perceptions of the Arabic-speaking community in Sydney, Australia toward the human papillomavirus (HPV) vaccination program: A qualitative study. *Vaccines.* 2021; 9(9 4 0):1–10.
58. (75) Schwartz DB. Integrating patient-centered care and clinical ethics into nutrition practice. *Nutrition in Clinical Practice.* 2013; 28(5):543–555. https://doi.org/10.1177/0884533613500507.
59. (77) Halal foods. Texas A&M University. https://animalscience.tamu.edu/wp-content/uploads/sites/14/2016/01/Halal-Foods.pdf. Published 2013. Accessed November 12, 2019.
60. (78) Carlowe J. Something for every-one. *Nursing Standard.* 2007; 22(4):20.
61. (79) Lee YY, Lin JL. Linking patients’ trust in physicians to health outcomes. *Br J Hosp Med.* 2008; 69(1):42–46..
62. (80) Islamic Organization of Medical Sciences. The Judicially Prohibited and Impure Substances in Foodstuff and Drugs. Paper presented at: The Judicially Prohibited and Impure Substances in Foodstuff and Drugs. 1995; Kuwait. https://www.immunize.org/talking-about-vaccines/porcine.pdf. Accessed September 15, 2019.
63. (81) Poku S. Medicines derived from animal sources. *The Rotherham NHS Foundation Trust*. Rotherham, UK: NHS; 2015.
64. (82) Hassanein M, Al-Arouj M, Hamdy O, et al. Diabetes and Ramadan: Practical guidelines. International Diabetes Federation (IDF), in collaboration with the Diabetes and Ramadan (DAR) International Alliance. (2017). Diabetes Research and Clinical Practice. 126:303–316. https://doi.org/10.1016/j.diabres.2017.03.003.
65. (83) Abolaban H, Al-Moujahed A. Muslim patients in Ramadan: A review for primary care physicians. *Avicenna Journal of Medicine*. 2017; 7:81–87. https://doi.org/10.4103/ajm.AJM_76_17.
66. (84) Ali NB, Nievea HR, Percac-Lima S, et al. Health care use by Muslim patients during Ramadan. Journal of Health Care for the Poor and Underserved. 2019; 30:1360–1372. https://doi.org/10.1353/hpu.2019.0099.
67. (85) Morris D. Managing diabetes in primary care during Ramadan. *Practice nursing*. 2020; 31(4):150–154.
68. (86) Beshyah SA, Hassanein M, Ahmedani MY, et al. Diabetic hypoglycaemia during Ramadan fasting: A *trans*-national observational real-world study. *Diabetes Research and Clinical Practice.* 2019; 150:315–321. https://doi.org/10.1016/j.diabres.2019.01.039.
69. (88) Alabbood MH, Ho KW, Simons MR. The effect of Ramadan fasting on glycaemic control in insulin dependent diabetic patients: A literature review. *Diabetes & Metabolic Syndrome: Clinical Research & Reviews.* 2017;11(1):83–87.
70. (89) Ahmed WN, Arun CS, Koshy TG, et al. Management of diabetes during fasting and COVID-19- Challenges and solutions*. Journal of Family Medicine and Primary Care*. 2020; 9:3797–806. https://doi.org/10.4103/jfmpc.jfmpc_845_20.
71. (90) Wallin AM, Lofvander M, Ahlstrom F. Diabetes: a cross-cultural interview study of immigrants from Somalia. *J Clin Nurs.* 2007; 16(11C):305–14. https://doi.org/10.1111/j.1365–2702.2007.02099.x.
72. (91) Al Zahrani AM, Zawawi MM, Almutairi NA, et al. The impact of Ramadan on visits related to diabetes emergencies at a tertiary care center. (2021). BMC Emergency Medicine 21:162. https://doi.org/10.1186/s12873-021–00555-8.
73. (92) Hui E, Bravis V, Salih S, et al. Comparison of Humalog Mix 50 with human insulin Mix 30 in type 2 diabetes patients during Ramadan. (2010). The International Journal of Clinical Practice. 64(8):1095–1099. https://doi.org/10.1111/j.1742–1241.2010.02347.x.
74. (93) Tourkmani AM, AlHarbi TJ, Bin Rsheed AM, et al. Impact of Ramadan Focused Education Program on medications adjustment for patients with type 2 diabetes in a primary health care institution in Saudi Arabia. (2019). Diabetes & Metabolic Syndrome: Clinical Research and Reviews. 13:161–165. https://doi.org/10.1016/j.dsx.2018.07.012
75. (94) Zainudin SB, Hussain AB. The current state of knowledge, perception and practice in diabetes management during fasting in Ramadan by healthcare professionals. (2017). Diabetes and Metabolic Syndrome: Clinical Research and Reviews. 12:337–342. https://doi.org/10.1016/j.dsx.2017.12.025.
76. (95) Yilmaz TE, Basara E, Tilmaz T, et al. Approaches and awareness of family physicians on diabetes management during Ramadan. (2021). International Journal of Clinical Practice. 75:e14205. https://doi.org/10.1111/ijcp.14205.
77. (96) Ali M, Adams A, Hossain MA, et al. Primary care providers’ knowledge and practices of diabetes management during Ramadan. (2016). Journal of Primary Care & Community Health. 7(1):33–37. https://doi.org/10.1177/2150131915601359.
78. (97) Hamodat H, Syed S, Ali M, et al. Primary care physicians’ knowledge, perceptions, and comfort level in managing patients fasting in Ramadan. (2020). Journal of Primary Care & Community Health. 11:1–5. https://doi.org/10.1177/215013270933796.
79. (98) Gaborit B, Dutour O, Ronsin O, et al. Ramadan fasting with diabetes: An interview study of inpatients’ and general practitioners’ attitudes in the South of France. (2011). 37:395–402. https://doi.org/10.1016/j.diabet.2010.12.010.
80. (99) Darko N, Dalloss H, Hadjiconstantinou, et al. Qualitative evaluation of A Safer Ramadan, a structured education programme that addresses the safer observance of Ramadan for Muslims with type 2 diabetes. (2020). Diabetes Research and Clinical Practice. 160:107979. https://doi.org/10.1016/j.diabres.2019.107979.
81. (1 0 0) Grindrod K, Alsabbagh W. Managing medications during Ramadan fasting. *Canadian Pharmacists Journal/Revue des Pharmaciens du Canada.* 2017;150(3):146–149.
82. (1 0 1) Aadil N, Houti IE, Moussamih S. Drug intake during Ramadan. *BMJ.* 2004;329(7469):778–782.
83. (1 0 2) Mughal F. Ramadan: What it means for general practice. (2014). British Journal of General Practice. https://doi.org/10.3399/bjgp14X680605
84. (1 0 3) Unal M, Ozturk O, Ozturk G, et al. Effect of fasting on smoking addiction. (2021). Journal of Addictions Nursing. https://doi.org/10.1097/JAN.0000000000000414.
85. (1 0 4) Khan ID, Khan SA, Asima B, et al. Morbidity and mortality amongst Indian Hajj pilgrims: A 3-year experience of Indian Hajj medical mission in mass-gathering medicine. (2018). Journal of Infection and Public Health. 1:165–170. https://doi.org/10.1016/j.jiph.2017.06.004.
86. (1 0 5) El-Bushra HE, Abodahish AA. Utilization of primary health care services during Hajj. (1999). Saudi Medical Journal. 20(12):931–938.
87. (1 0 6) Alqahtani AS, Alsharif SA, Garnan MA, et al. The impact of receiving pretravel health advice on the prevention of Hajj-related illnesses among Australian pilgrims: Cohort study. (2020). JMIR Public Health and Surveillance. 6(3):e10959. https://doi.org/10.2196/10959
88. (1 0 7) Alqahtani AS, Sheikh M, Wiley K, et al. Australian Hajj pilgrims’ infection control beliefs and practices: Insight with implications for public health approaches. (2015). Travel Medicine and Infectious Disease. 13:329–334. https://doi.org/10.1016/j.tmaid.2015.05.009.
89. (1 0 8) Sridhar S, Belhouchat K, Drali T, et al. French Hajj pilgrims’ experience with pneumococcal infection and vaccination: A knowledge, attitudes and practice (KAP) evaluation. (2015). Travel Medicine and Infectious Disease. 13:251–255. https://doi.org/10.1016/j.tmaid.2015.02.002.
90. (1 0 9) Gawthrop M. Advising Hajj and Umrah travellers in general practice. (2020). Practice Nursing. 31(4):156–161.
91. (1 1 1) Mughal F, Chew-Graham CA, Saad A. Hajj: What it means for general practice. (2018). British Journal of General Practice. https://doi.org/10.3399/bjgpopen18X101493.
92. (1 1 2) Inayat Q. Islamophobia and the therapeutic dialogue: Some reflections. (200&). Counselling Psychology Quarterly. 20(3):287–293.
93. (1 1 3) Dallo FJ, Prabhakar D, Ruterbusch J, et al. Screening and follow-up for depression among Arab-Americans. (2018). Depression and Anxiety:351198–1206. https://doi.org/10.1002/da.22817.
94. (1 1 4) Guerin B, Guerin P, Diiriye RO, et al. Somali conceptions and expectations concerning mental health: Some guidelines for mental health professionals. (2004). New Zealand J of Psychology:33(2)59–67.
95. (1 1 5) Mölsä M, Tiilikainen M, Punamaki RL. Usage of healthcare services and preference for mental healthcare among older Somali immigrants in Finland. (2019). Ethnicity & Health. 24(6):607–622. https://doi.org/10.1080/13557858.2017.1346182.
96. (1 1 6) Amri S, Bemak F. Mental health help-seeking behaviors of Muslim immigrants in the United States: Overcoming social stigma and cultural mistrust. (2012). Journal of Muslim Mental Health, 7(1):43–63.
97. (1 1 7) Killawi A, Daneshpour M, Elmi A, et al. Recommendations for promoting healthy marriages & preventing divorce in the American Muslim community. (2014) Institute for Social Policy and Understanding.
98. (1 1 8) Koenig HG, Peteet JR, VanderWeele TJ. Religion and psychiatry: clinical applications. (2020). BJPysch Advances. 25:273–281. https://doi.org/10.1192/bja.2020.11.
99. (1 1 9) Haque A, Khan F, Keshavarzi H, et al. Integrating Islamic traditions in modern psychology: Research trends in last ten years. (2016). Journal of Muslim Mental Health 10(1).
100. (1 2 0) Michlig GJ, Johnson-Agbakwu C, Surkan PJ. “Whatever you hide, also hides you”: A disclosure analysis on mental health and service use in an American community of Somalis. (2022). Social Science & Medicine. 292:114563. https://doi.org/10.1016/j.socscimed.2021.114563.
101. (1 2 1) Haroun Z, Bokhari A, Marko-Holguin M, et al. Attitudes toward depression among a sample of Muslim adolescents in the Midwestern United States. (2011). International J Adolescent Med Health. 23(3):293–301. https://doi.org/10.1515/IJAMH.2011.058.
102. (1 2 2) Shah ZA. On Organ Donation and Transplantation. The Fiqh Council of North America. https://fiqhcouncil.org/on-organ-donation-and-transplantation/. Published 2018. Accessed December 22, 2019.
103. (1 2 3) Albar M. Organ Transplantation: A Sunni Islamic Perspective. *Saudi Journal of Kidney Diseases and Transplantation.* 2012;23(4):817–822.
104. (1 2 8) Orr RD, Genesen LB. Requests for “inappropriate” treatment based on religious beliefs. (1997). Journal of Medical Ethics. 23:142–147.
105. (1 2 9) Teno JM, Gruneir A, Schwartz Z, Nanda A, Wetle T. Association between advance directives and quality of end‐of‐life care: A national study. *Journal of the American Geriatrics Society.* 2007;55(2):189–194.
106. (1 3 0) Ashana DC, D/Arcangelo N, Gazarian PK, et al. “Don’t talk to them about goals of care”: Understanding disparities in Advance Care Planning. (2022). J Gerontol A Biol Sci Med Scie. 77(2):339–346. https://doi.org/10.1093/Gerona.glab091.
107. (1 3 1) Tulsky JA. Beyond advance directives: importance of communication skills at the end of life. *JAMA.* 2005;294(3):359–365.
108. (1 3 2) Boucher NA, Siddiqui EA, Koenig HG. Supporting Muslim patient during advanced illness. (2017). The Permanente Journal. 21:16–190. https://doi.org/10.7812/TPP/16–190.
109. (1 3 3) Dy SM, Isenberg SR, Abu Al Hamayel N. Palliative care for cancer survivors. (2017). Medical Clinics of North America. 101:1181–1196. https://doi.org/10.1016/j.mcna.2017.06.009.
110. (1 3 4) Mathew-Geevarughese SE, Corzo O, Figuracio E. Cultural, religious, and spiritual issues in palliative care. (2019). Primary Care Clinical Office Practice. 46:399–413. https://doi.org/10.1016/j.pop.2019.05.006.
111. (1 3 5) Biondo PD, Kalia R, Khan RA, et al. Understanding advance care planning within the South Asian community. (2017). Health Expectations. 20:911–919. https://doi.org/10.1111/hex.12531.
112. (1 3 6) Ahmed SM, Lemkau JP. Cultural issues in the primary care of South Asians. (2000). Journal of Immigrant Health. 2(2): 89–96.
113. (1 3 7) Miller AC, Ziad-Miller A, Elamin EM. Brain death and Islam: The interface of religion, culture, history, law, and modern medicine. *Chest.* 2014;146(4):1092–1101.
114. (1 3 8) Arbour R, AlGhamdi HM, Peters L. Islam, brain death, and transplantation: Culture, faith, and jurisprudence. (2012). AACN Advanced Critical Care. 23(4):381–394. https://doi.org/10.1097/NCI.0b013e3182683b1e.
115. (1 3 9) Ebrahim AFM. Islamic jurisprudence and the end of life. (1998). Medicine and Law. orr17(2):189–196.
